# Difficult Biliary Cannulation for Intradiverticular Papilla: Forceps Technique Revisited

**DOI:** 10.1055/s-0041-1731442

**Published:** 2021-08-03

**Authors:** Mahesh Kumar Goenka, Gajanan Ashokrao Rodge, Bhavik Bharat Shah, Shivaraj Afzalpurkar

**Affiliations:** 1 Institute of Gastrosciences & Liver, Apollo Gleneagles Hospital, Kadapara, Phool Bagan, Kankurgachi, Kolkata, West Bengal, India

**Keywords:** periampullary diverticula, ERCP, two-devices in one-channel method, difficult biliary cannulation

## Abstract

Periampullary diverticula (PAD) have been encountered in 5.9 to 18.5% of patients during all the endoscopic retrograde cholangiopancreatography (ERCP). Cannulation in the presence of PAD can sometimes be difficult, time consuming, and often requires a higher level of endoscopic skills.

Several techniques have been reported to facilitate and increase the chances of successful bile duct cannulation in the presence of PAD. The two-devices in one-channel method has been sparingly used. It involves the simultaneous use of a biopsy forceps and another instrument, either a cannula or sphincterotome through the same working channel. We successfully performed ERCP in three cases, where bile duct cannulation was performed in the setting of intradiverticular papilla using two-devices in one-channel method.

We feel that the two-devices in one-channel method can be very useful and positioned higher up in the algorithm for successful cannulation in patients with PAD.

Periampullary diverticula (PAD) have been encountered in 5.9 to 18.5% of patients during all the endoscopic retrograde cholangiopancreatography (ERCP). 1 2 These are categorized according to the location of the major papilla in relation to the diverticulum. 2 3 Cannulation in the presence of PAD can sometimes be difficult, time consuming, and often requires a higher level of endoscopic skills. Several techniques such as submucosal saline injection, endoclip-assisted cannulation, reversed guidewire method, double endoscope method, cap-assisted cannulation, entering the duodenal diverticulum, and dilation of the diverticular neck by balloon have been reported to facilitate and increase the chances of successful bile duct cannulation in the presence of PAD. 4 5 6 7 8 9 10 11

The two-devices in one-channel method has been sparingly used. This method involves the simultaneous use of a biopsy forceps and another instrument, either a cannula or a sphincterotome through the same working channel. 12 13 While the biopsy forceps is used to bring the papilla out of the diverticulum and stabilize it, biliary cannulation is attempted with the sphincterotome or cannula. Since both the devices enter the same channel of the therapeutic duodenoscope, it is named as two-devices in one-channel method. We successfully performed ERCP in three cases ( Table 1 ), where bile duct cannulation was performed in the setting of intradiverticular papilla using two-devices in one-channel method.

**Table 1:** Summary of patient characteristics

Sr. no.	Age	Sex	Diagnosis	Position of papilla in relation to PAD	Method used	Successful CBD cannulation	Adverse events
1	78	Male	Choledocholithiasis with recurrent cholangitis in postcholecystectomy status	7 o'clock	Two-devices in one-channel method	Yes	None
2	54	Female	Choledocholithiasis with obstructive jaundice	5 o'clock	Two-devices in one-channel method	Yes	None
3	52	Male	EHBO with mass in uncinate process and head of pancreas	7 o'clock	Two-devices in one-channel method	Yes	None

Abbreviations: CBD, common bile duct; EHBO, extrahepatic biliary obstruction; PAD, periampullary diverticulum.

## Case Series

### Case 1

**Fig. 1:**
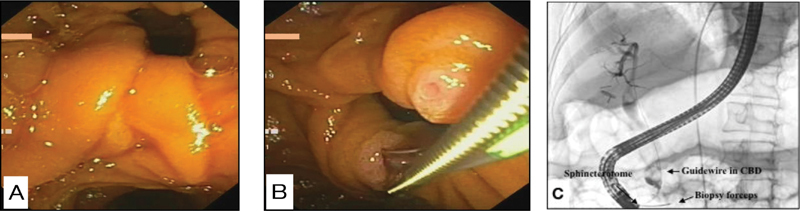
( **A** ) Side view endoscopy showing a large periampullary diverticulum. Papilla could not be identified. ( **B** ) Sphincterotome and biopsy forceps being used simultaneously to explore the papilla and facilitate cannulation. ( **C** ) Fluoroscopy image showing biliary cannulation by simultaneous use of biopsy forceps and sphincterotome.

A 78-year-old male presented with complaints of fever, jaundice, and pain abdomen and was diagnosed to have cholangitis with choledocholithiasis. He was referred to our center after a failed attempt at ERCP cannulation. He was a known case of type 2 diabetes mellitus, systemic hypertension, obstructive airway disease, chronic kidney disease, dilated cardiomyopathy, and status post cholecystectomy. At the time of admission, the patient was afebrile and hemodynamically stable. Laboratory parameters revealed a hemoglobin (Hb) of 9.9 g/dL, white blood cell counts were 4,500 /mm ^3^ , and platelets were 1,30,000/mm ^3^ . Liver function tests showed total bilirubin of 2.8 mg/dL, aspartate transaminase (AST) of 273 U/L, alanine transaminase (ALT) of 294 U/L, and alkaline phosphatase (ALP) of 343 U/L. Multiple intraluminal calculi at the distal end of the common bile duct (CBD) with proximal dilatation were seen at magnetic resonance cholangiopancreatography (MRCP) ( Fig. 1A and 1B ).

The patient was managed conservatively with intravenous antibiotics and other supportive measures after admission. At ERCP, a therapeutic duodenoscope (channel diameter 4.2 mm) was used, and a large PAD (around 1.5 cm in diameter) was seen in side view endoscopy ( Fig. 1A ). The papillary orifice could not be visualized for biliary cannulation. A pediatric biopsy forceps (diameter—2 mm) was used to grasp the inferior lip of the diverticulum, and the diverticular mucosa was pulled out, exposing the papilla, which was located inside the diverticulum at 7 o'clock position. The sphincterotome (diameter—1.8 mm) was used simultaneously, and successful biliary cannulation was achieved ( Fig. 1B ). Cholangiogram of the biliary system revealed a filling defect in the CBD ( Fig. 1C ) after which endoscopic sphincterotomy and stone extraction were performed. Postprocedure was uneventful and the patient was asymptomatic for 3 months on follow-up.

### Case 2

A 54-year-old female, known case of type 2 diabetes mellitus, hypothyroidism, chronic kidney disease, and coronary artery disease, presented with pain abdomen, jaundice, and vomiting for 2 weeks. At the time of admission, the patient was hemodynamically stable, afebrile, and icterus was present. Laboratory parameters revealed a Hb of 6.5 g/dL, white blood cell counts were 10,100/mm ^3^ , and platelets were 90,000/mm ^3^ . Liver function tests showed total bilirubin of 9.4 mg/dL (direct bilirubin of 5.9 mg/dL), AST of 46 U/L, ALT of 9 U/L, ALP of 328 U/L, and gamma-glutamyl transpeptidase (GGT) of 164 U/L. MRCP showed chronic calculous cholecystitis and choledocholithiasis with dilated intrahepatic biliary radicles.

**Fig. 2:**
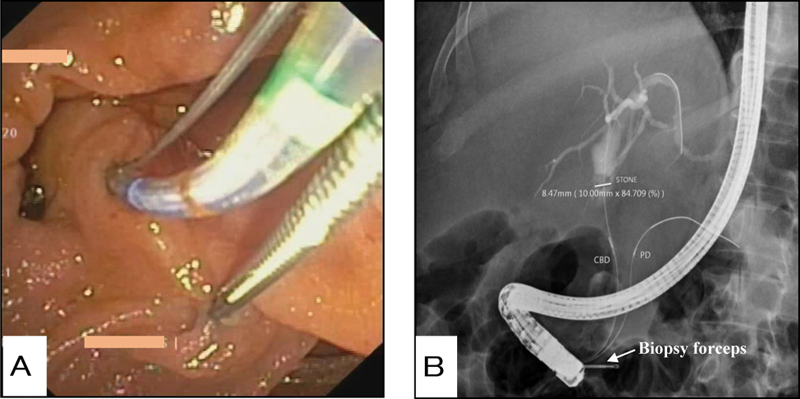
( **A** ) Sphincterotome and biopsy forceps being used simultaneously leading to exposure of papilla and successful cannulation. ( **B** ) Fluoroscopy image showing pancreatic duct-assisted biliary cannulation by simultaneous use of biopsy forceps and sphincterotome.

During ERCP, a large PAD (around 2 cm in diameter) was seen with the papillary orifice at 5 o'clock position in relation to the diverticulum. Two-devices in one-channel method, as described above, was used and biliary cannulation was attempted by simultaneous use of pediatric biopsy forceps and sphincterotome ( Fig. 2A ). Guidewire inadvertently entered in the main pancreatic duct (MPD), after which pancreatic duct assisted CBD cannulation was done. Cholangiogram of the biliary system revealed dilated CBD with a filling defect ( Fig. 2B ), after which endoscopic sphincterotomy and removal of stone were performed. A plastic stent was deployed in PD (5 Fr × 5 cm) and another plastic stent was positioned in the CBD (10 Fr × 10 cm). Postprocedure was uneventful and the patient was asymptomatic for 3 weeks on follow-up.

### Case 3

A 52-year-old male, known case of type 2 diabetes mellitus, presented with jaundice and fever for the last 2 weeks. On admission, the patient was hemodynamically stable, febrile and icterus was present. Laboratory parameters showed a Hb of 12.4 g/dL, white blood cell counts were 9,200/mm ^3^ and platelets were 5,94,000/mm ^3^ . Liver function tests showed total bilirubin of 12.5 mg/dL (direct bilirubin of 7.5 mg/dL), AST of 105 U/L, ALT of 120 U/L, ALP of 1,297 U/L, and GGT of 807 U/L. Carbohydrate antigen 19-9 levels were 220 U/mL. Computed tomography abdomen revealed an ill-defined hypodense mass in the head and uncinate process of pancreas with dilated CBD and MPD, likely carcinoma head of the pancreas.

At ERCP, a PAD was visualized with the papillary orifice at 7 o'clock position in relation to the diverticulum. Two-devices in one-channel method as described in the first case was used, and biliary cannulation was attempted with the simultaneous use of pediatric biopsy forceps and sphincterotome. Cholangiogram of the biliary system revealed stricture in lower CBD with upstream dilatation. Brush cytology was taken from the stricture area. Biliary sphincterotomy was done, and a 6 cm fully covered self-expanding metal stent was deployed across the stricture. Subsequent liver function tests showed a downward trend. Brush cytology from CBD did not show any malignant cells and subsequent endoscopic ultrasound-guided fine needle aspiration from the pancreatic head mass was suggestive of adenocarcinoma. Postprocedure was uneventful, and the patient was asymptomatic for 3 weeks on follow-up.

## Discussion

ERCP in patients with PAD requires the use of specialized techniques and an experienced endoscopist. Out of the 410 cases who underwent ERCP during the study duration, PAD was seen in 34 cases. Cannulation was achieved successfully by the standard technique in 31 cases with PAD; however, despite 10 minutes of efforts, cannulation was not achieved in three patients for whom the two-devices in one-channel method was used.

**Fig. 3:**
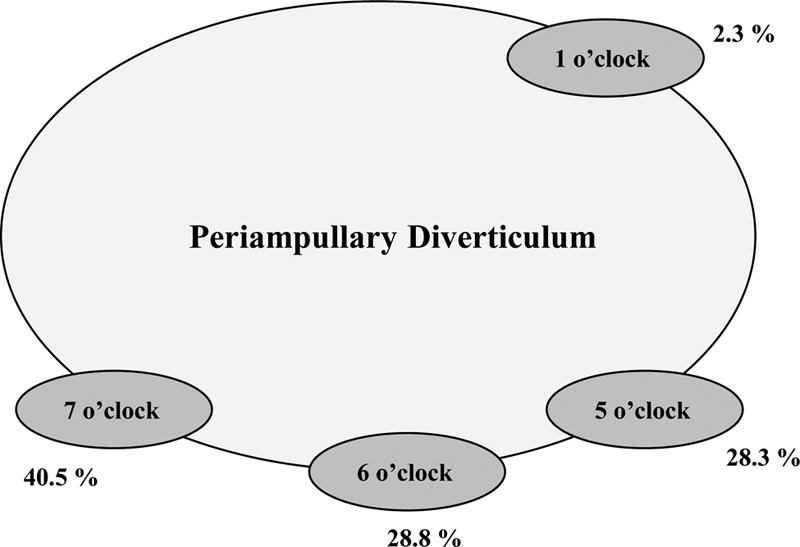
Different locations of the papilla in relation to the diverticulum. (Adapted and modified from Parlak et al. 2 )

Ease of cannulating the papilla also depends on the position according to the diverticulum. Fig. 3 shows the different locations of the papilla in relation to the diverticulum. 2 Cannulation procedures are more difficult in the 1 o'clock position as compared with the other locations of the papilla. The rates of successful cannulation reported in PAD patients vary from 61 to 95.4%, which are markedly lower than that seen in patients without PAD. 14

Patients with PAD are more likely (1.8–6 times) to have retained CBD stones as compared with those without PAD. 15 16 The possible factors leading to increased formation of biliary stones in the presence of PAD are dysfunction in the sphincter of Oddi, 17 diverticula causing spasm of the sphincter, and increased biliary tract pressure 18 or diverticula, leading to biliary stasis by compression of the distal part of the CBD. 19

Several methods have been described in the literature to facilitate difficult biliary cannulation in the presence of PAD that has been summarized in Table 2 . The method to be used counts on the endoscopist's choice and patient conditions. The central goal of the different techniques is to bring the papilla in a better position and angle, suitable for cannulation. We have successfully used the two-devices in one-channel method in three cases with PAD. We feel that this can be a very useful technique that can be positioned higher up in the algorithm for successful cannulation in patients with PAD.

**Table 2:** ERCP techniques to facilitate biliary cannulation in presence of PAD

Sr. no.	Technique	Devices used	Remarks
**1**	PD stent placement followed by precut sphincterotomy	PD stent precut needle knife	Stent placement keeps papilla out of the diverticulum
**2**	Submucosal saline injection	Saline injector	2–4 mL of normal saline is injected in the submucosa that enables intradiverticular papillary eversion
**3**	Endoclip-assisted cannulation	Endoclips	Endoscopic clips evert and stabilize the papillary opening
**4**	Reversed guidewire method	Reversed guidewire (stiff end forward)	Reverse end of the guidewire used to push papillary mucosa toward the lumen and thus everting the papilla
**5**	Double endoscope method	Duodenoscope, gastroscope, FB forceps	Grasping tissue adjoining the papilla with forceps through the gastroscope followed by insertion of the duodenoscope
**6**	Cap-assisted cannulation	Transparent cap, papillotome	Papillary orifice is directly seen using cap-assisted forward viewing endoscopy
**7**	Entering the diverticulum	Duodenoscope	Enter the duodenal diverticulum with distal end of the duodenoscope
**8**	Dilatation of the diverticular neck by balloon	Stone retrieval balloon	Intradiverticular balloon dilation allows eversion of the papilla
**9**	Two-devices in one-channel method	Biopsy forceps, catheter (cannula, sphincterotome)	Simultaneous use of biopsy forceps and sphincterotome

Abbreviations: ERCP, endoscopic retrograde cholangiopancreatography; FB, foreign body; PAD, periampullary diverticulum; PD, pancreatic duct.

## References

[1] Fogel E L Sherman S Lehman G A Increased selective biliary cannulation rates in the setting of periampullary diverticula: main pancreatic duct stent placement followed by pre-cut biliary sphincterotomy Gastrointest Endosc 1998 47 05 396 400 9609434 10.1016/s0016-5107(98)70226-3

[2] Parlak E Suna N Kuzu U B Diverticulum with papillae: does position of papilla affect technical success? Surg Laparosc Endosc Percutan Tech 2015 25 05 395 398 25730737 10.1097/SLE.0000000000000130

[3] Boix J Lorenzo-Zúñiga V Añaños F Domènech E Morillas R M Gassull M A Impact of periampullary duodenal diverticula at endoscopic retrograde cholangiopancreatography: a proposed classification of periampullary duodenal diverticula Surg Laparosc Endosc Percutan Tech 2006 16 04 208 211 16921297 10.1097/00129689-200608000-00002

[4] Harada H Suehiro S Shimizu T Katsuyama Y Hayasaka K Submucosal injection can facilitate biliary access in patients with periampullary diverticula Gastrointest Endosc 2016 84 01 185 186 26772894 10.1016/j.gie.2016.01.002

[5] Cappell M S Mogrovejo E Manickam P Batke M Endoclips to facilitate cannulation and sphincterotomy during ERCP in a patient with an ampulla within a large duodenal diverticulum: case report and literature review Dig Dis Sci 2015 60 01 168 173 25138902 10.1007/s10620-014-3321-1

[6] Kim H J Kim Y S Myung S J A novel approach for cannulation to the ampulla within the diverticulum: double-catheter method Endoscopy 1998 30 09 S103 S104 9932770 10.1055/s-2007-1001432

[7] Elmunzer B J Boetticher N C Reverse guidewire anchoring of the papilla for difficult cannulation due to a periampullary diverticulum Gastrointest Endosc 2015 82 05 957 26142553 10.1016/j.gie.2015.05.054

[8] Külling D Haskell E Double endoscope method to access intradiverticular papilla Gastrointest Endosc 2005 62 05 811 812 16246708 10.1016/j.gie.2005.06.035

[9] Myung D S Park C H Koh H R Cap-assisted ERCP in patients with difficult cannulation due to periampullary diverticulum Endoscopy 2014 46 04 352 355 24549783 10.1055/s-0034-1365060

[10] Wang B C Shi W B Zhang W J Entering the duodenal diverticulum: a method for cannulation of the intradiverticular papilla World J Gastroenterol 2012 18 48 7394 7396 23326150 10.3748/wjg.v18.i48.7394 PMC3544047

[11] Tóth E Lindström E Fork F T An alternative approach to the inaccessible intradiverticular papilla Endoscopy 1999 31 07 554 556 10533741 10.1055/s-1999-59

[12] Fujita N Noda Y Kobayashi G Kimura K Yago A ERCP for intradiverticular papilla: two-devices-in-one-channel method Gastrointest Endosc 1998 48 05 517 520 9831843 10.1016/s0016-5107(98)70096-3

[13] Inamdar S Stein P H Han D Trindade A J Sejpal D V Difficult biliary cannulation achieved in the setting of periampullary diverticulum with the simultaneous use of biopsy forceps and wire-guided cannulation VideoGIE 2017 2 02 25 26 29905244 10.1016/j.vgie.2016.12.008 PMC5990488

[14] Zoepf T Zoepf D S Arnold J C Benz C Riemann J F The relationship between juxtapapillary duodenal diverticula and disorders of the biliopancreatic system: analysis of 350 patients Gastrointest Endosc 2001 54 01 56 61 11427842 10.1067/mge.2001.115334

[15] Rajnakova A Goh P M Ngoi S S Lim S G ERCP in patients with periampullary diverticulum Hepatogastroenterology 2003 50 51 625 628 12828047

[16] Mohammad Alizadeh A H Afzali E S Shahnazi A ERCP features and outcome in patients with periampullary duodenal diverticulum ISRN Gastroenterol 2013 2013 217261 23984079 10.1155/2013/217261 PMC3747500

[17] Yildirgan M I Başoğlu M Yilmaz I Periampullary diverticula causing pancreaticobiliary disease Dig Dis Sci 2004 49(11-12):1943 1945 15628730 10.1007/s10620-004-9597-9

[18] Hagège H Berson A Pelletier G Association of juxtapapillary diverticula with choledocholithiasis but not with cholecystolithiasis Endoscopy 1992 24 04 248 251 1612038 10.1055/s-2007-1010476

[19] Miyazaki S Sakamoto T Miyata M Yamasaki Y Yamasaki H Kuwata K Function of the sphincter of Oddi in patients with juxtapapillary duodenal diverticula: evaluation by intraoperative biliary manometry under a duodenal pressure load World J Surg 1995 19 02 307 312 7754640 10.1007/BF00308647

